# LATE WORKING LIFE IN THE TRIPLE TRANSITION AND THE GLOBAL PERMACRISIS

**DOI:** 10.1093/geroni/igad104.1787

**Published:** 2023-12-21

**Authors:** Andreas Motel-Klingebiel, Gerd Naegele, Gulin Oylu, Indre Genelyte, Susanne Kelfve

**Affiliations:** Linkoping University, Norrköping, Ostergotlands Lan, Sweden; Institut fur Gerontologie, Dortmund, Nordrhein-Westfalen, Germany; Linkoping University, Linkoping, Ostergotlands Lan, Sweden; Linkoping University, Linkoping, Ostergotlands Lan, Sweden; Linköping University, Norrköping, Ostergotlands Lan, Sweden

## Abstract

Late working life faces multiple challenges in the triple transition of demographic, technological and environmental change and the global permacrisis. This contribution provides a conceptual framework for research on the significance of extended late work for the sustainability, equity, inclusion, and productivity of aging societies, based on an understanding of the three major long-term shifts as a complex triple transition with potentials and challenges for societies, economies, employers, and individuals. This triple transition is embedded in an ongoing and even accelerating societal and economic permacrisis with increased social risks and limited predictability, in which resilience – of economies, life-courses and welfare states - gains importance, while the dominant tetrad of sustainability, equity, inclusion and productivity remains on the European social and economic policy agenda. The presentation introduces a review of European policy reports on aging and late work and introduces results from the research programme EIWO (www.eiwoproject.org) based on Swedish register information and European survey data to illustrate trends and gaps in European late work, research, and policy. The presentation discusses age integration of life and work courses as a systematic strategy to promote quality of life as well as social sustainability, equity, inclusion and productivity and resilience. Participants will gain an understanding of the conceptualization and implementation of gerontological multi-level research on the macro- and micro-social relevance of late working life in the triple transition and the global permacrisis.

